# Herbo-mineral ayurvedic treatment in a high risk acute promyelocytic leukemia patient with second relapse: 12 years follow up

**DOI:** 10.4103/0975-9476.72618

**Published:** 2010

**Authors:** Balendu Prakash, Purvish M. Parikh, Sanjoy K. Pal

**Affiliations:** *V C P Cancer Research Foundation (Scientific & Industrial Research Organization), Dehradun, India*; 1 *AmeriCares India, Khar (West), Mumbai, India*; 2 *IPCA Traditional Remedies Pvt Ltd., Mumbai, India*

**Keywords:** Ayurveda, herbo-mineral, relapse acute promyelocytic leukemia

## Abstract

A 47 year old diabetic male patient was diagnosed and treated for high risk AML-M3 at Tata Memorial Hospital (BJ 17572), Mumbai in September 1995. His bone marrow aspiration cytology indicated 96% promyelocytes with abnormal forms, absence of lymphocytic series and myeloperoxide test 100% positive. Initially treated with ATRA, he achieved hematological remission on day 60, but cytogenetically the disease persisted. The patient received induction and consolidated chemotherapy with Daunorubicin and Cytarabine combination from 12.01.96 to 14.05.96, following which he achieved remission. However, his disease relapsed in February 97. The patient was given two cycles of chemotherapy with Idarubicine and Etoposide, after which he achieved remission. His disease again relapsed in December 97. The patient then refused more chemotherapy and volunteered for a pilot Ayurvedic study conducted by the Central Council for Research in Ayurveda and Siddha, New Delhi. The patient was treated with a proprietary Ayurvedic medicine Navajeevan, Kamadudha Rasa and Keharuba Pisti for one year. For the subsequent 5 years the patient received three months of intermittent Ayurvedic treatment every year. The patient achieved complete disease remission with the alternative treatment without any adverse side effects. The patient has so far completed 13 years of survival after the start of Ayurvedic therapy.

## INTRODUCTION

Acute promyelocytic leukemia (APL) is a unique subtype of the acute leukemias. It has distinct cytogenetics, clinical features, and biologic characteristics. APL is caused by arrest of leukocyte differentiation at the promyelocyte stage. The discovery and elucidation of the molecular pathogenesis for APL has led to the first and only targeted therapy for leukemia. APL is classified as AML M3 in the French-American and British (FAB) classification and represents approximately 5-10% of all leukemia in adults.[1] Indian data on this malignancy are however, limited.[2] In about 95-98% of cases, APL is associated with the reciprocal translocation, t(15; 17)(q22; q21) and the reciprocal fusion of the retinoic acid receptor (RAR)α gene on chromosome 17 with the promyelocytic leukemia (PML) gene on chromosome 15. The resulting PML-RARα fusion gene encodes a chimeric protein that causes the pathogenesis of APL.[1] Diagnosis of the disease is made by examining the bone marrow. The typical phenotype of acute promyelocytic leukemia (APL) is myeloperoxidase positive and CD33 positive, human leukocyte antigen (HLA)-DR negative. The disease is said to be in remission when bone marrow study reveals no cluster or collection of blast cells, and normal maturation of all cellular components (i.e. erythrocytic, granulocytic, and megakaryocytic series). There are less than 5% blast cells in the bone marrow and none have a leukemic phenotype (e.g. Aner rods). The persistence of dysplasia is an indicator of residual disease. The absence of a previously detected clonal cytogenetic abnormality (i.e. complete cytogenetic remission) confirms the diagnosis of complete remission.

Acute myeloid leukemia (AML) is a curable disease; the chance of cure for a specific patient depends on a number of prognostic factors.[3] The single most important prognostic factor in AML is cytogenetics, or the chromosomal structure of the leukemic cell. Certain cytogenetic abnormalities are associated with very good outcomes (e.g., the (15;17) translocation in APL). About half of AML patients have normal cytogenetics; they fall into an intermediate risk group. A number of other cytogenetic abnormalities are known to associate with a poor prognosis and a high risk of relapse after treatment.[4] For patients with relapsed AML, the only proven potentially curative therapy is a stem cell transplant, if one has not already been performed. Patients with relapsed AML who are not candidates for stem cell transplantion, or who have relapsed after a stem cell transplant, may be offered treatment in a clinical trial, as conventional treatment options are limited.

The introduction of all-trans retinoic acid (ATRA) and, more recently, arsenic trioxide (ATO) into the therapy of APL has revolutionized the management and outcome of this disease. Several treatment strategies using these agents, usually in combination with chemotherapy, but also without or with minimal use of cytotoxic agents, have provided excellent therapeutic results.[5] Current state-of-the-art treatment, which include simultaneous administration of ATRA and anthracycline-based chemotherapy for induction and consolidation, as well as ATRA-based maintenance, have dramatically transformed APL into the most curable acute leukemia in adults, with approximately 80% of long-term survivors.[6] However, about 5%-30% of patients relapse, mainly patients with high-risk APL.[7] Treatment of relapsed/advanced APL includes the use of arsenic trioxide (ATO), gemtuzumab ozogamicin, and hematopoietic stem cell transplantion. ATO is currently the most effective therapeutic agent in relapsed APL. However, current therapeutic results are still unsatisfactory in untreated patients and poorer in those with primary refractory or relapsed disease.

Here we describe the case of a forty-seven year old patient successfully treated with oral herbo-mineral Ayurvedic formulations viz. Keharuba Pisti, Kamadudha Rasa, and Navajeevan.[8] after relapsing twice with conventional therapy. This case was part of a pilot study conducted by the Central Council for Research in Ayurveda and Siddha (CCRAS)[9] New Delhi in 1997. The pilot study observed complete disease remission in 11/11 APL patients who completed 90 days of Ayurvedic treatment.

## METHODOLOGY

No direct reference to leukemia or its sub classifications has been definitely identified in the Ayurvedic literature, though some scholars identify present day leukemia with *Raktarbuda/ Raktapitta* since certain symptoms of leukemia resemble those given for these diseases. Leukemia is a clonal, neoplastic proliferation of immature cells of the haemopoetic system, which are classified by aberrant or arrested differentiation. Leukemic cells rapidly accumulate in the bone marrow cavity replacing most of normal haemopoetic cells, resulting in signs and symptoms of disease. The rationale for using Ayurvedic medicines is to restore homeostasis and reverse the proliferation of neoplastic cells in the bone marrow. Details of the Ayurvedic medicines are given below: [Tables 1–3]:

Dose and administration of drug

**Table 1:** Composition of Navajeevan* Anupan: Water

Traditional Name	English / Scientific Name	Proportion
Rajat Bhasma	Silver Bhasma	1 part
Jaharmohra	Serpentine stone	1 part
Nirvisha	*Delphinium denudatum*	1 part
Taruni, gulab	*Rosa centifolia*	1 part
Chandan	*Santalum album*	1 part
Gojihva	*Onosma bracteatum*	1 part
Lata kasturi	*Hibiscus abelmoschus*	1 part

* Proprietary Ayurvedic Medicine

**Table 2:** Composition of Kamadudha Rasa Anupan: Mishri

Traditional Name	English / Scientific Name	Proportion
Mauktik Pishti	*Mytilus margaritiferus preparation*	1 part
Pravala pisti	*Corallium rubrum preparation*	1 part
Mukta sukti pisti	*Mytilus margaritiferus*	1 part
Kapardika bhasma	*Calcinated and purified Cypraea moneta shells*	1 part
Sankha bhasma	*Calcinated and purified Turbinella rapa shells*	1 part
Svarna gairika	*Calcinated and purified Ochre*	1 part
Amrta satva	*Tinospora cordifolia extract*	1 part

**Table 3:** Composition of Keharuba Pisti

Trinakanta Mani churna – 1 part Gulab arka - Q. S. (for mardana)

1. Navjeevan tablet:
  First three months - 250mg tablet three times daily
  Next nine months - 125mg tablet three times daily
2. Kamdudha Ras (250mg) + Kehruba Pisti (125mg)
  First three months - four times daily mixed with honey
  Next nine months - Only Kamdudha (250 mg) three times daily

## CASE REPORT

A weak and febrile 47 years old diabetic male presented to Tata Memorial Hospital (TMH) on 04.09.95 with hemoglobin 5.6 gm% and total count 17,100/cu mm. Investigation of the case at TMH confirmed diagnosis of AML M3. His Bone Marrow Aspiration (BMA) cytology indicated diluted cellularity; myeloid series – promyelocytes 96%; lymphocytic series and megakaryocytes were absent. Cytochemistry: a myeloperoxidase test was 100% positive. The patient was started with ATRA for 90 days from 14.09.95, after which he developed bronchospasm, high grade fever and odynophagia. He was treated with blood components and antibiotics conservatively. Bone marrow analysis carried out on 13.11.95 indicated that cellularity, erythropoesis and myelopoesis were normal. However, 1% lymphopoesis blasts were present. His induction chemotherapy was given from 12.01.96 to 18.01.96. Post chemotherapy the patient had fever and vomiting; these problems were managed conservatively. The patient then received 3 cycles of consolidation chemotherapy with Daunorubicin and Cytarabine combination from 12.01.96 to 14.05.96. The patient achieved clinical remission; however, cytogenetically the disease persisted. At initial diagnosis the patient was 100% t(15;17) positive and after (ATRA + 4 cycle chemo) he was 50% t(15;17) positive. For the next seven months the patient was relatively event free. In January 97 the patient again presented to THM for checkup. Investigations done at TMH indicated relapse of the disease. BMA indicated cellularity – hypocellular; erythropoesis – hyperplasia; myelopoesis – blast 2%, abnormal promyelocytes 75%, myelocytes 10% (NEC blast was seen), mature myelocytes 3%, band 10%. The patient then underwent two cycles of chemotherapy with Idarubicine and Etoposide. The first and the second cycle were given from 18.02.97 and 18.05.97, respectively. The patient tolerated the chemotherapy with some side effects. For the next 6 months after the completion of chemotherapy the patient was in remission. The Bone marrow investigation on 12/12/97 indicated a second relapse of the disease. Blast cell 14%, abnormal promyelocytes 85% (Faggot cells present) and 1% basophil were seen. A cytochemistry report found a myeloperoxidase test to be 100% positive. The patient was again advised to undergo another round of chemotherapy with a combination of Mitoxantrone, Cytarabine and Etoposide. However, he refused further chemotherapy and volunteered for a pilot study conducted by the Central Council for Research in Ayurveda and Siddha (CCRAS),[7] and try an Ayurvedic therapy advocated by the first author. The patient’s bone marrow slide from TMH was reviewed at Institute of Rotary Cancer Hospital (AIIMS), New Delhi, and he was enrolled for the study on 22.12.97.

When the Ayurvedic therapy started, the patient was very weak, anemic, anorexic, febrile, had a lung infection and a big rectal abscess. His hemoglobin was 7.0 gm%, total count was 1050/cu mm, neutrophil to lymphocyte ratio was 5:95. The patient was treated conservatively with antibiotics (oral, I.V. and local application), along with Ayurvedic therapy. He was also given blood transfusion and human granulocyte colony-stimulating factor to improve his WBC count. Strict isolation was maintained, calorie rich diet devoid of *Pittavardhak aahar* and salt was prescribed. After about 15 days of alternative therapy the patient’s general condition started to improve, his lung infections subsided and he started to maintain normal body temperature. After 1 month of therapy his differential counts started to show a normal picture. As the patient continued with the therapy his weakness gradually subsided. The frequency of blood transfusion was also reduced. Before the start of Ayurvedic therapy he had received 269 units of blood components viz. 20 units of fresh frozen plasma; 32 units of packed cells; 31 units of whole blood; 165 units of platelets concentrate and 21 units of plasma concentrate from 07.09.95 to 18.12.95. After the start of Ayurvedic therapy the patients received 16 units of whole blood; 1 unit of packed cell and 6 units of plasma concentrate in the first two months of the therapy. No further blood transfusion was required. The Ayurvedic therapy was continued for one year and stopped. BMA studies done after 90 days Ayurvedic treatment on 01.04.98 indicated the disease was in remission. Though there was mild erythroid hyperplasia, granulocytic series appeared to be normal in morphology and maturation. No abnormal cells were seen. For the subsequent 5 years till 2003 the patient received three months Ayurvedic treatment every year. Subsequent, BMA performed on 19.01.2000 and 14.11.2000, respectively from Bangalore Institute of Oncology, Bangalore and Sir Ganga Ram Hospital, New Delhi detected no abnormality. Details of the hematological parameters investigated during follow up are given in Table 4. During the treatment period, and later during follow up, the patient was constantly monitored for liver and kidney function. The Ayurvedic treatment did not produce any hepato or renal toxicity. In June 2002 the patient had purpuric lesions on both legs with itching and burning sensation. His problems were managed conservatively. At present the patient has completed 7 years of relapse free survival after the stoppage of Ayurvedic therapy. He is still in follow up and doing well along with conventional management for his diabetes.

**Table 4:** Details of hematological and bone marrow study of the patients

Date	HB g/dl	TLC cu/mm	Differential leukocyte count	Plt Cu/mm (lakhs)	ESR mm/1 hr
			Neutrophil/ Lymphocyte/Eosinophil/ Monocyte / Basophil		
04.09.95	Admitted to Tata Memorial Hospital (TMH), Mumbai. APL diagnosis confirmed. Cytogenetic analysis of peripheral blood cells showed t(15:17). Started on ATRA for 90 days. Induction and consolidation complete in TMH on 25.05.96. Complete remission of the disease achieved.				
20.05.96	6.5	8000	53/43/03/01/0	0.11	
14.02.97	Relapse of the disease; Second round of chemotherapy was given which was completed in June 97; BMA after chemotherapy indicated complete remission.				
12.12.97	Bone marrow aspiration: Relapse of the disease; Blast 14% Abnormal promyelocytes 85% (Faggot cells seen), Basophil 1%.				
08.01.98	6.0	59000	35/30/05/08/0/ Blast 15/ Promyelocytes 16	0.8	
01.04.98	Bone marrow aspiration: No abnormal cells seen. Investigation done from Bangalore Institute of Oncology.				
31.01.99	16.7	6950	49/41/08/02/0	1.3	12
19.01.00	Bone marrow biopsy and aspiration: Marrow in remission. Investigation done from Bangalore Institute of Oncology.				
08.11.00	16.0	9500	64/30/04/02/0	1.8	18
14.11.00	Bone marrow aspiration: Findings are suggestive of hematological remission. Investigation done from Sir Ganga Ram Hospital, New Delhi.				
22.11.01	16.0	8400	70/24/06/0/0	1.8	10
07.11.02	15.8	8300	60/30/01/09/0	1.7	44
13.12.03	16.5	6300	59/33/07/01/0	1.58	
06.11.05	14.0	6000	54/42/03/01/0	2.10	25
06.06.07	13.8	6900	65/26/04/05/0	2.05	10
13.05.09	13.3	5300	67/30/02/01/0	1.59	65

HB – hemoglobin; TLC – Total leukocyte count; Plt – Platelet count; ESR – Erythrocyte sedimentation rate.

## DISCUSSION

The case presented here indicates that Ayurvedic medicines are effective in treatment of APL. The same medicines also produced promising results in many other leukemia patients who could not afford regular treatment.[10] Encouraging results had been previously observed in the pilot study conducted by CCRAS.[9]

Significant numbers of leukemia patients in India try various systems of complementary and alternative medicine (CAM),[11] of which, Ayurveda is the most commonly used. The present study indicates that the chosen Ayurvedic medicines were effective in the treatment of APL and did not produce any toxic side effects. As the patient did not receive any other therapy after his second relapse, the Ayurvedic therapy can be considered responsible for the remission of his disease. The many APL patients who relapse after undergoing best of conventional therapy have limited options. An Ayurvedic approach like the one given here can be of some help to them. However, proper clinical trials are needed to substantiate our observations so that beneficial alternative therapies can be integrated with conventional care.
